# When All Else Fails, Listen to the Patient: A Viewpoint on the Use of Ecological Momentary Assessment in Clinical Trials

**DOI:** 10.2196/11845

**Published:** 2019-04-21

**Authors:** Aaron M Mofsen, Thomas L Rodebaugh, Ginger E Nicol, Colin A Depp, J Philip Miller, Eric J Lenze

**Affiliations:** 1 Department of Psychiatry School of Medicine Washington University in St Louis St Louis, MO United States; 2 Department of Psychological and Brain Sciences Washington University in St Louis St Louis, MO United States; 3 Department of Psychiatry University of California - San Diego San Diego, CA United States; 4 Division of Biostatistics School of Medicine Washington University in St Louis St Louis, MO United States

**Keywords:** ecological momentary assessment, mental health, controlled clinical trial, psychiatry, health technology

## Abstract

A major problem in mental health clinical trials, such as depression, is low assay sensitivity in primary outcome measures. This has contributed to clinical trial failures, resulting in the exodus of the pharmaceutical industry from the Central Nervous System space. This reduced assay sensitivity in psychiatry outcome measures stems from inappropriately broad measures, recall bias, and poor interrater reliability. Limitations in the ability of traditional measures to differentiate between the trait versus state-like nature of individual depressive symptoms also contributes to measurement error in clinical trials. In this viewpoint, we argue that ecological momentary assessment (EMA)—frequent, real time, in-the-moment assessments of outcomes, delivered via smartphone—can both overcome these psychometric challenges and reduce clinical trial failures by increasing assay sensitivity and minimizing recall and rater bias. Used in this manner, EMA has the potential to further our understanding of treatment response by allowing for the assessment of dynamic interactions between treatment and distinct symptom response.

## Introduction

### Background

Mental health treatment development and testing has been at an impasse for the past several decades; our clinical trials increasingly fail more often than in other fields [1]. Although the global burden of psychiatric illness continues to be one of the largest contributors to disability worldwide, investment in the discovery of novel pharmacologic agents flows instead toward disease states with identifiable biological targets. These targets remain elusive in psychiatric disorders [2,3]. The central nervous system (CNS) drug development pipeline has become increasingly burdened with late-phase failures [4], contributing to a well-publicized exodus of the pharmaceutical industry from the CNS space. This has resulted in decreased investment in drug discovery [5].

### Treatment Failures: Bad Medicine or Bad Measures?

The randomized, placebo-controlled trial is still considered the gold standard test of treatment efficacy. However, over the past 60 years of treatment research in psychiatry, we have observed that treatment effect sizes remain stable, whereas placebo responses rise [6]. Modern clinical trials are difficult to conduct and are fraught with numerous challenges related to cost, regulatory requirements, recruitment difficulties, and other inefficiencies [7,8]. Added to these challenges is the use of imprecise outcome measures, which hinders the ability to detect true separation of active treatment from placebo response [9].

The contribution of poor measures to treatment failures is particularly well-illustrated in antidepressant trials [10-12]. For example, lanicemine, an N-methyl-D-aspartate receptor antagonist differing from ketamine that produces lower psychotomimetic side effects, was thought to show promise in treating depression [13]. Early phase clinical trials showed promising results in rapidly reversing symptoms of treatment resistant depression, but investigators failed to replicate the results in a late phase study [14]. Similarly, basimglurant, a postsynaptic metabotropic glutamate subtype 5 receptor antagonist, showed promise in early phase trials but failed to separate from placebo on the primary outcome measure in a larger phase 2b trial [15]. In both cases, the primary end point was change from baseline to 6 weeks in the Montgomery Asberg Depression Scale (MADRS), which is considered an industry standard in depression treatment research. The authors identified flaws in study design, conduct, and even underlying scientific rationale as possible causes of these late stage failures.

It seems unlikely, given the financial and intellectual resources brought to bear in the early phases of discovery, that investigators could have gotten the scientific rationale so wrong. A more probable explanation for the failed studies might lie in how the primary outcome was determined and measured. Although the MADRS is considered a standard assessment tool in depression research, poor interrater reliability (ie, imprecision of measurement) is one of many limitations to this measure’s assay sensitivity.

### The Culprit: Faulty Signal Detection

Measurement *assay sensitivity*, as it applies to clinical research, refers to the ability of a symptom assessment measure to detect whether a difference exists between treatment groups [16]. Issues of assay sensitivity are well known in psychiatric treatment research and have been observed with older self-report scales such as the Hamilton Rating Scale for Depression (HAM-D) as well as in newer clinician-administered instruments such as the MADRS. Both measures include several symptom domains but offer only a final summed score. This offers little insight into the specific symptoms underlying the clinical presentation.

Self-report measures may incorporate reporter bias, whereas clinician-administered assessments incorporate bias on the part of the clinician. For example, there may be bias in recruitment or sample ascertainment, such as career patients who serially enroll in research studies for financial reasons and are thus motivated to answer questions in such a way as to increase likelihood of enrollment. Investigators may unconsciously inflate baseline measures of psychiatric symptoms to meet recruitment goals [17-19].

Nonetheless, these arguments fail to explain why academic studies, in which less financial gain accrues to the patient and investigator, also see a high placebo response and failure rate [20]. Regardless, reduced assay sensitivity in clinical trials has the potential to sabotage treatment development at any stage. We submit that these and other depression symptom measures reduce assay sensitivity in 3 primary ways: unnecessary complexity, human error (ie, clinician judgment), and infrequent sampling.

### Getting to Precision Assessment

The idea of using technology to increase the accuracy and precision of symptom assessment in clinical trials is gaining momentum. For example, the National Institutes of Health toolbox was designed specifically for this purpose [21]. The Patient-Reported Outcomes Measurement Information System also offers researchers standardized patient-reported outcome (PRO) measurement tools with transparent performance metrics [22]. Self-report measures delivered via mobile technology certainly offer ecological validity and may also prove superior to clinician-administered instruments in large, industry-funded clinical trials. Improved measurement would likely translate into more useful clinical trials. It may even go a long way toward surmounting our present impasse in developing new mental health treatments.

Clearly, we are not the first to contemplate the problem of assay sensitivity in our field. However, public discussion as to why progress in the field of psychometrics has stalled has not extended to industry trials. Open scientific discourse has also been limited on the subject of developing novel, effective, Food and Drug Administration (FDA)–sanctioned instruments, which could be used to track mental health disorder outcomes with greater assay sensitivity. As the success or failure of antidepressant treatment trials often rests solely on the presumed validity and reliability of symptom measures, it should follow that these assessments deserve the same degree of scrutiny regarding assay sensitivity as any laboratory test.

In this viewpoint, we will examine 3 major problem areas we believe the field needs to address in getting to precision assessment: overly complex assessment tools, contributions of human error, and limitations of infrequent sampling. First, we will review the 2 gold standard depression instruments used at present to track psychiatric symptoms in industry-funded drug trials. Next, we will examine the role of clinician assessment and how human involvement in measurement contributes to error. We will then discuss challenges to adequate measurement frequency in obtaining valid self-report data. Finally, we propose a solution to the measurement problem in depression clinical trials. We will explore contributions from the fields of mathematics, human psychology, and computer science to the development of mobile technology–based measures, which we believe may offer significant improvements over traditional symptom assessment.

## Problem 1: Needless Complexity Undermines Utility

Key point:

- Overly broad measures that attempt to cover multiple symptoms or symptom domains compromise signal detection. To meaningfully reduce error, consensus on what to measure is needed.

### The Problem of Excessive Description

Psychiatric rating scales frequently use diagnostic criteria or descriptive psychopathology to track a patient’s progress throughout a clinical trial. The descriptive psychopathology for a given psychiatric disorder is by nature more expansive than the diagnostic criteria alone, which can be helpful for identifying clinically significant features for treatment targets. This problem is not restricted to mental health research; trials in cardiology have also been compromised by failing to adequately confine outcome measures for meaningful signal detection [23]. In major depression, patients often have irritability, anxiety, and other symptoms in addition to the 9 cardinal symptoms of the disorder. A content analysis by Eiko Fried found 52 symptoms of depression across 7 commonly used depression scales, with a content overlap among all scales of only 32 percent [24].

Take for example the MADRS discussed above [25]. The clinician in using this scale administers a 10-item assessment to a study participant. The change in the total score over time is then used to determine whether the treatment under investigation is effective. The 17-item HAM-D (HAM-D-17) determines efficacy similarly [26]. However, both items assess multiple symptom domains, all considered diagnostic aspects of depression. A recent study by Checkroud et al [27] of over 7000 patients with major depression demonstrates why this approach, as well as any other that relies on indiscriminate use all of the items in a scale to assess primary efficacy outcomes (eg, the HAM-D), may be a problem. In their study, they illustrate how this indiscriminate approach to measurement can jeopardize a potential treatment in late-phase clinical trials. Specifically, they found that consistent antidepressant treatment response was found only for the core emotional symptoms (anergia, dysphoria, anhedonia, feelings of worthlessness, and difficulty concentrating). The detectable signal for treatments shown to be effective is thus obscured by the total score, which is the only score considered when designing trials to determine efficacy. This example highlights how standard rating scales have contributed to treatment failures by introducing unnecessary *complexity*, which reduces measurement specificity.

To further complicate matters, measuring multiple constructs inflates the chance that items tied to each construct will shift unpredictably over time (eg, due to lack of longitudinal factorial invariance) [28]. In this way, depression rating scales are often a mix of sensitive and specific items (dysphoria, anhedonia), nonspecific items (anxiety), and symptoms that may be derived from an unrelated illness (eg, fatigue). Side effects of the treatment itself are also frequently conflated with the items in the primary outcome measure. Moreover, individual items within a scale are often not weighted for relevance. As the success or failure of a treatment rests on a scale’s summative score, it follows that some of the score’s equally weighed items might be totally irrelevant to the trajectory of the disorder in question [29]. The 24-item HAM-D (HAM-D-24) is more comprehensive than the 17-item version [30]. It was designed to more comprehensively capture relevant symptoms. However, using the HAM-D-24 may conceal treatment effects by introducing items that assess uncommon or diagnostically nonspecific symptoms, such as hypochondriasis or depersonalization. Again, as the total score is used to determine whether or not a treatment is effective, there is a further risk of magnifying irrelevant changes and obscuring important ones.

### Less is More

The shortened 6-item HAM-D and MADRS scales, which favor core items such as low mood, anhedonia, and guilt, have both been shown to be more sensitive than HAM-D-17 and the 10-item MADRS, respectively [31]. The shorter 6-item version of the HAM-D [32] was superior to the longer HAM-D-17, 21 and 24 in detecting treatment response to the newer antidepressant vortioxetine versus placebo [33]. Similarly, the buprenorphine/samidorphan combination treatment, which failed to separate from placebo on the primary outcome measure of change from baseline on the MADRS-10 item scale, fared better in separating from placebo using the MADRS-6 item scale [34]. These examples suggest a data reduction approach to symptom assessment focusing on core symptoms is more likely to accurately detect meaningful clinical response. Unfortunately, there is, as of yet, little agreement on which symptoms are most relevant.

Consensus on the most clinically, functionally, or personally relevant features of treatment response or remission is needed to improve signal detection. If we simply wish to use our existing scales more pragmatically, we would take a treatment we know to be effective and choose the individual items from a selected scale that reveal the greatest amount of separation in favor of the proven treatment. We would then use the items from that same scale to determine whether or not an unproven treatment is effective. Alternatively, the field could adopt a universal consensus around measuring the core emotional symptoms of the illness to determine treatment success or failure. This is a difficult and unlikely scenario as we do not have the evidence base at present necessary to establish what exactly these core symptoms might be. In either case, improvement from a functional or pharmacoeconomic perspective may not map well onto any of the items in the measures we currently use. This may force the field to revisit some of its a priori assumptions about clinical relevance. In short, although we can confidently say that our current approach is suboptimal, fixing it will not be so easy.

## Problem 2: Human Error Magnifies Measurement Error

Key points:

- Clinician-administered scales compound response bias
- Self-report alone is imperfect but minimizes rater contribution to measurement error

### Not All That Glitters is Gold

Psychiatric treatment research has traditionally considered clinician-administered assessments to be the *gold standard* over PRO measures. This stems in part from an inherent belief that the clinician *objectively corrects* for whatever error (eg, errors of omission, exaggeration, expectancy effect, and Hawthorne effect), intentional or otherwise, introduced by the patient. Perhaps somewhat counterintuitively, clinicians may *magnify* the patient’s error. A large study evaluating self-report and clinician-administered instruments from the Sequenced Treatment Alternatives to Relieve Depression trial found that self-report measures contributed more to the prediction of outcomes of clinician-administered instruments than vice versa [35]. The authors of the study also recommended that, in the event that only 1 form of assessment could be used, self-reported outcome measures would be preferable.

Error or bias on the part of the clinician is routine, rather than idiosyncratic. It would be unfair to presume it to be the result of malice or laziness. It may happen unconsciously and even in good faith because clinical judgment is not completely objective. Interviewers are also susceptible to either a positive or negative rater bias depending on whether research participant attributes, often irrelevant to the assessment at hand, are perceived as positive or negative. This can result in sometimes pronounced unconscious alterations of judgment [36] that significantly impact clinical decision making. This has been illustrated in studies finding poor interrater and test-retest reliability in standard clinician-administered assessment measures for depression [3]. The reason for such results may be that clinicians, even when given rules governing the scoring of the assessment at hand, will tend to drift from standard calibrated practice [37]. Whether or not a clinician reliably follows an assessment-related rule depends on the amount of inertia that must be overcome to adopt it, the format in which the rule was originally presented, the number of demands that compete with the rule, and the institutional pressures involved in maintaining compliance with the rule [38].

### When all Else Fails, Listen to the Patient

Although the evidence is still far from conclusive, a decent body of literature has elevated the stature of PROs vis-a-vis traditional, clinician-administered rating scales. Self-report assessments represent an improvement over clinician-administered assessments insofar as they eliminate rater bias and reduce the likelihood that participants will feel compelled to give socially desirable responses (a type of response bias) or affirmative answers when interviewed face-to-face [39]. For example, a large meta-analysis of placebo response in 96 antidepressant trials by Mora et al found that clinician-administered instruments were associated with a higher placebo response than PRO measures [40]. Such evidence further supports the idea that clinician-administered scales add error rather than removing or mitigating patient error. In summary, although we place a high value on clinician-administered assessments, clinician objectivity may be more of an appealing myth than reality.

## Problem 3: Infrequent Sampling Hurts Sensitivity

Key points:

- Retrospective patient symptom report in the context of a clinical trial may be inaccurate
- Ecologically valid symptom reports collected in real time are needed to interpret treatment effects

### (Not So) Total Recall

Self-report also has inherent limitations. This was recognized by Arthur Schopenhauer in the 19th century [41], who observed that one cannot be both the subject and object of accurate perception. Thus, reporting on one’s own mood even in the present poses significant challenges and represents an irremediable layer of error. Mehl and Conner have also comprehensively discussed the problem of recall bias in psychological research [42]. In short, asking a participant to provide a retrospective symptom report merely compounds this error by introducing recall bias. In other words, emotional recall bias (unlike the subject-object problem) is a controllable source of error. Neuroscientists have found memory to be frequently unreliable, particularly when the encoding and retrieval of memories occurs during periods of emotional arousal [43]. Memory has many odd biases, not all of which are evident in daily life. For instance, it has been shown that people have a tendency to remember events that ought to be enjoyable, such as a vacation or spending time with one’s children, as being more pleasant than they actually were [42]. Thus, asking a respondent to recall something requires filtration through whatever emotional state the subject happens to be in at the time of the assessment, which only compounds this error [44]. Furthermore, respondents are unlikely to accurately create a coherent summary of their emotional states over time.

### What is the (Right) Frequency?

Infrequent measurement or sampling in clinical trials tacitly makes the assumption that we know enough about how an illness behaves over time to ask questions with a time frame modifier (eg, “In the last week...”) and is associated with measurement error in clinical trials. This has been illustrated in disciplines outside of psychiatry. For example, the Heart Outcomes Prevention Evaluation trial evaluated the effect of the angiotensin-converting enzyme inhibitor ramipril in patients at high risk for adverse cardiovascular events [45]. The study found that ramipril lowered blood pressure assessed via 24-hour ambulatory measurement, whereas office-based blood pressure measurements did not detect the treatment response. Investigators attributed this to a diurnal variation in blood pressure or *white coat hypertension* —phenomena that could not be captured with the limited number of measures obtained during office hours or that were affected by the office visit itself. For this reason, blood pressure assessment in clinical trials has moved to using frequent ambulatory blood pressure sampling to assess treatment efficacy, which has essentially eliminated the placebo response in antihypertensive treatment trials [46,47].

Similar to blood pressure, depressive symptoms also appear to fluctuate throughout the day or in response to specific situations [48]. Mobile technology offers a feasible way to increase sampling frequency, as evidenced by the already rich scientific literature on ambulatory assessment [42]. However, this approach has yet to be fully embraced by industry sponsored studies, where it could be of prime utility. To date, only 1 industry-sponsored study currently underway has attempted to compare daily, ambulatory self-report with a clinician-administered measure [49]. Frequent, in-the-moment self-report also has its limitations. There is no doubt some theoretical limit on high-frequency sampling to the extent that it may, if administered often enough, conflate mood and emotions or succeed in becoming itself a source of negative mood, affect, or emotions [50,51]. However, this issue calls for careful experimentation with frequency to assess acceptability rather than avoiding frequent sampling altogether.

### The State Versus Trait Problem

Symptoms of many psychiatric illnesses are characterized as trait-like in advance of any evidence to support this assumption. However, variation is routinely observed in behaviors studied over time, irrespective of how trait-like they seemed to be (eg, personality traits such as sociability) [52]. For this reason, it is highly probable that important variation is the rule rather than the exception in psychiatric illness. For example, in an individual with major depression, mood might be very depressed at a certain point in the morning and near-normal later that same day [48].

Despite this, we continue to measure mood as a stable trait-like symptom (eg, “in the last 7 days, how has your mood been?”). This is the case for most psychiatric symptom assessments, where dynamic versus stable or trait-like nature of symptoms are poorly described. The only way to ascertain variation or lack thereof is to sample the illness frequently *before finalizing the measure (eg, for use in a treatment study).* In other words, frequent sampling would ideally be used to inform the creation of a scale before using it to track efficacy [52]. Without this approach, scale selection becomes thoughtlessly reflexive [50]. Limited sampling likely further compromises psychiatric research because trait measures require respondents to attempt a summation of states via recall of past experiences, which has been shown to introduce error [53].

Even if the symptoms of psychiatric illness are predominantly trait-like, we would continue to favor frequent sampling, even if this requires us to use a smaller number of items. This is in contrast to classical test theory, from which we take the maxim that adding equally good items to a measure leads to greater reliability and therefore, a better shot at validity [54]. This is based on the ideal circumstance where it is possible to ask a respondent the same question repeatedly, which we cannot do at a single time point without expecting the respondent to become reactive to the question [54,55]. Furthermore, a measure using high-signal items repeatedly over time would better capture any given quality than would a measure with a mix of items with lower signal detection at a single time point [56]. In psychiatric treatment research, we have historically chosen to use a greater number of inferior items at a single time point, even though the maxim we are following was based on equations that are arguably better suited to repeated measurement of a single quality.

## Solution: Ecological Momentary Assessment

### Overview

Ecological momentary assessment (EMA) is frequent, real time, patient-reported assessment delivered via surveys (eg, “right now, my mood is...”) and completed by the patient typically via mobile device to collect information about the patient in a real-world setting [57]. Participants are prompted at prespecified intervals to complete symptom assessments rather than having a prompt dependent upon a passive event (eg, actigraphy and patterns of speech). EMA may overcome the deficiencies inherent in traditional clinician-administered instruments. Evidence from pain studies examining EMA alongside retrospective recall show a consistent discrepancy between the 2 forms of report [58]. A similar discrepancy between real time and retrospective self-report of affect has also been demonstrated [59]. A single item scale measuring mood delivered via EMA outperformed the HAM-D-17 in its ability to predict “current relapse status” in patients with major depressive disorder [60].

### Increasing Accuracy in Early Phase Trials

Frequent, real-time EMA sampling has been shown in the same study to both qualify positive findings in clinical trials and detect treatment effects that the HAM-D was unable to detect between groups after 18 weeks of treatment [61]. Frequent real-time sampling has also been shown to unmask differences between treatment responders and nonresponders and to detect treatment effects earlier than clinician-administered assessments [62,63]. Finally, frequent, real-time sampling compared with retrospective assessment has been shown to increase the precision of measurement over time.

An example of how infrequent sampling adversely affects assay sensitivity in clinical trials was recently provided by Moore et al [64]. In this study, the researchers assessed the effects of mindfulness-based stress reduction (MBSR), compared with an attention placebo. For outcome assessments, they measured depressive symptoms, anxiety symptoms, and mindfulness self-ratings in 2 ways: EMA tools delivered to participants electronically via a smartphone 3 times daily for 14 days and traditional paper- and pencil-based measurement tools asking about last week’s symptoms (comparable with most outcome measures). The EMA-based outcome assessment resulted in a much lower number needed to treat (NNT) for MBSR than the same outcomes measured using the traditional technique: the NNT for treating depression was 8 using EMA versus 31 using traditional measurement. In other words, EMA captured a treatment effect that was missed by standard self-report assessments. This was also reflected in the smaller SDs for outcomes measured via EMA when averaged over time. In short, frequent ambulatory assessment improves precision.

### Increased Understanding of Core Symptom Constructs

EMA may also increase measurement precision by tracking how symptoms of an illness behave and interact over time [65]. This allows investigators to characterize state versus trait-like symptoms and establish the nature of the relationships between symptoms over time. This approach may also be useful because it offers the ability to evaluate interactions between symptoms without first assuming that they are symptoms *of the disorder in question.* This “pragmatic nihilism” [66] or “symptomic” [67] approach differs from how we currently assess psychiatric disorders. Clinician-administered instruments are rated with the built-in assumption that any number of symptoms are all tied to 1 underlying, latent variable (eg, depression). With enough patient-reported EMAs carried out over time, investigators may be able to observe how symptoms interact with one another.

It may also be possible to discern which symptoms are central to the disorder under study and how certain upstream symptoms may influence a cascade of symptoms downstream. How many EMAs are *enough* depends on the exact questions being asked and the assumptions made in the analysis; however, it is likely that as little as 25 measurements from hundreds of participants or a hundred measurements in even a small number of participants would be a reasonable starting place [68]. Such findings may eventually afford researchers the unique opportunity to stratify clinical trial participants based on *how* they do or do not get better rather than simply whether or not they get better. The approach becomes highly descriptive at the level of the individual, thereby allowing one to answer a host of previously unanswerable questions.

### Deconstructing Treatment Response

Another question that might be asked is whether patients responding to an intervention or placebo get better in the same way. In other words, do the *temporal dynamics* of placebo response differ from that observed in drug response? Temporal dynamics here refer to certain discernable patterns in the EMA data that allow a researcher to broadly classify a patient as displaying, for instance, affective inertia (symptoms strongly relate to themselves over time, resulting in less change over time), affective instability (symptoms vary a great deal over time), or inability to differentiate between symptoms (as 1 symptom gets better or worse the rest tend to follow) [69]. This is by no means an exhaustive list of questions that may be asked of the data derived from EMA. It is safe to say EMA has the potential to offer a renaissance of sorts in descriptive psychopathology and may even allow for veritable *personalized medicine* given the types of patterns and points of intervention it is able to reveal.

EMA may also help us detect the phenomenon of regression to the mean. This phenomenon occurs when a baseline assessment of symptoms in a clinical research study is inflated at the initial visit before regressing to where those symptoms normally *live*. This is thought to significantly impact the ability to detect separation whenever it occurs in the placebo group. Using EMA, patients may be monitored in the outpatient setting not simply for clinical research purposes but rather to give the clinician a better idea of whether or not a patient is getting better. This approach appreciates EMA as an instrument that may be used to conduct field research, which is thought to have better “ecological validity” than assessments delivered within the artificial environment of the clinical trial site [42]. Such real-world information could be used to find out where that patient “lives” if a patient is being screened for a clinical research study. Similarly, it is not difficult to envision tailoring inclusion/exclusion criteria to this end. If and when this does take place, CNS research will be indebted to data provided directly by the patient.

### Developing Better Interventions

Once individual symptom characteristics are known, targeted interventions can be developed. For instance, if insomnia leads to anergia the following day, which in turn leads to anhedonia, one might examine whether applying an intervention at the onset of insomnia changes the observed course of symptomatology downstream. This sort of intervention is called an ecological momentary intervention (EMI) because it relies on EMA or a just-in-time adaptive intervention. An EMI is an intervention informed by data gathered by EMA. We can already find examples of researchers using EMA data to provide an EMI. For example, EMI has already been shown to be very successful in providing patients with substance use disorders relapse prevention tools precisely when they need it the most [70]. It is conceivable that EMA scales, in addition to providing efficacy outcomes with increased assay sensitivity, may also reveal novel points of intervention in clinical trials.

Multiple methods, including multilevel vector autoregression and multilevel dynamic structural equation modeling, can help researchers examine how individuals may vary from group trends over time [71,72]. This might allow clinicians to tailor a personalized EMI based on a patient’s own unique pattern of EMA data. To take this idea further still, EMA may eventually be able to offer the unique ability to evaluate whether a target is being addressed by an intervention via *real-time* lagged mediation rather than post hoc analyses. In other words, we would be able to use real-time lagged mediation to see whether or not we are actually engaging a chosen target precisely when we are attempting to target it.

The use of EMA to gather the data needed to deliver a just-in-time EMI is also consistent with the concept of target engagement raised by the National Institute of Mental Health in an effort to address the declining success of clinical trials in mental health. A target is defined as something “molecular, cellular, circuit, behavioral or interpersonal, commensurate with the intervention,” which is expected to be changed in some way by the intervention being studied [73]. The concept of target engagement is closely related to a recent call for a research focus on symptomics or the examination of “symptom-specific effects” [70]. Such a focus, as represented in the example above, may allow us to identify those key symptoms that tend to precede or perhaps even cause other symptoms. Investigating patterns of interaction between symptoms in this way may help us to understand some of the underlying causes of complex psychiatric illnesses.

## How Do We Get to Widespread Use of Ecological Momentary Assessment in Clinical Trials?

### Understanding and Getting Past Limitations

Although smartphone ownership is not universal, it is increasing, particularly among individuals with psychiatric conditions. John Torous found in a recent survey of 457 individuals with schizophrenia or schizoaffective disorder that greater than half (54%) of such individuals owned a smartphone [74]. Perhaps a greater question then is whether a participant with a smartphone would want to use it to regularly quantify his or her depressive symptoms. User privacy is also becoming an increasingly important issue as faith in *big tech* to safeguard users’ privacy has waned in the wake of the numerous scandals. Getting around these limitations may require sponsors to invest in low-cost devices participants can use while enrolled in trials.

Use of EMA in the real world often leads to missing data that have historically made analysis problematic. Users may not be compliant with the number of surveys they are required to complete in a timely manner, and, as described above, frequency of assessments increase precision only up to a point. Beyond this point, with too frequent assessment, the risk increases of either introducing noise by sampling irrelevant aspects of the human condition or of the assessment itself becoming a negative part of the intervention. Investigators will have to consider an assay sensitivity assessment as part of the startup process to determine how the target population will best respond to EMA.

Although the FDA has made its expectations for PRO measures clear [75], it is not at all clear whether every aspect of FDA guidance will neatly translate to electronic PROs. For example, to what extent, if any, would necessary software updates for an accepted EMA app involve the FDA? FDA guidance for evaluating antidepressant drugs has not been updated since 1977 and explicitly favors selecting scales that have been previously used in drug trials over ones that are novel [76]. This effectively prioritizes tradition over innovation and creates a catch-22 for researchers who might otherwise break with the status quo. Clinician-administered instruments need to be evaluated alongside commensurate EMA-delivered items. This will help us to determine parameters such as the optimal sampling frequency but will likely also be necessary as the FDA typically reports correlation coefficients for established measurement tools [77].

The conceptualization of disorders based on Diagnostic and Statistical Manual of Mental Disorders/International Classification of Diseases criteria has been called into question and may eventually be replaced altogether by Research Domain Criteria [78]. Although EMA is in many ways conducive to a dimensional approach to mental illness, this migration would obviously require a new approach to EMA scale creation and validation. In this case, the role of EMA may be to supplement observable behaviors with self-report.

EMA may not be ideal for detecting rare events, especially if they occur infrequently relative to the sampling frequency (ie, as the sampling frequency decreases so too does the probability of capturing *rare events*). Thus, when and how to apply EMA in clinical trials remains an area requiring additional study and consensus development.

EMA should not be mistaken for a panacea so long as p-hacking, publication bias, and alpha inflation continue to affect the integrity of clinical research. Any scale used to evaluate the efficacy of an intervention in large industry-sponsored clinical trials must be uniform and well-validated. Thus, to create a standard efficacy measure for a given psychiatric disorder, we first must form a consensus about the types of items that should be included in the EMA scales, the frequency and duration of assessments, and the types of analytical approaches that will be used to interpret the data. The FDA would be unlikely to accept an EMA-based primary outcome measure over existing efficacy end point measures without standardization across multiple field trials in different populations. These data should then clearly establish test-retest reliability, external validity, and other parameters necessary to validate an EMA scale.

### Conclusions

Moving from clinician-administered rating scales toward real-time patient-reported measures such as EMA offers significant advantages across medical settings. In clinical research studies, EMA may reduce placebo response and increase intervention-placebo separation. EMA also offers an obvious advantage over clinician-administered rating scales in inpatient and community settings given that time, cost, and staff pressures make use of the latter measure impractical. In community and inpatient settings, EMA can be used to identify individual factors leading to relapse, provide a more accurate picture of how a patient has been doing between clinical visits, and link real-world functional outcome measures over time (eg, rates of rehospitalization, days lost because of disability, and likelihood of self-harm) to *scores* on EMA scales. Finally, interventions are rapidly being introduced and delivered via smartphone. EMA may offer the best way to assess intervention acceptability and efficacy, creating the opportunity to personalize treatments with real-time adaptation. For these reasons, EMA is poised not only to replace clinician-administered rating scales in research settings but also to increase accessibility of EMA measures to the patients and health care providers in clinical settings, ultimately allowing real-world clinical settings to contribute meaningful data to research and development of new interventions.

Overall, we believe that the continued use of clinician-administered retrospective self-report assessments in clinical trials contributes significantly to observed treatment failures and squanders innovative potential. As we have described, the instruments currently being used are too broad to adequately assess outcomes, suffer from poor interrater reliability, make inappropriate assumptions about how the illness being studied behaves, and rely on patient recall despite a sizeable body of research, which cautions against this. EMA instruments may play an increasingly important role in addressing the disparity between the need for and investment in novel mental health treatments. Self-report assessment via EMA addresses the limitations of traditional assessment methods but has not yet made its way into large multisite clinical trials sponsored by the industry. Although the FDA’s recent efforts to advance mobile technology in clinical trials [79] represents an important first step, iterative testing of standardized EMA-delivered instruments to assess primary outcomes in clinical research is still needed.

## Abbreviations

CNS: central nervous system
EMA: ecological momentary assessment
EMI: ecological momentary intervention
FDA: Food and Drug Administration
HAM-D: Hamilton Rating Scale for Depression
HAM-D-17: 17-item Hamilton Rating Scale for Depression
HAM-D-24: 24-item Hamilton Rating Scale for Depression
MADRS: Montgomery Asberg Depression Scale
MBSR: mindfulness-based stress reduction
NNT: needed to treat
PRO: patient-reported outcome
